# Supplementary material for: Cardiac Hemangiomas: A Five-Year Systematic Review of Diagnosis, Treatment, and Outcomes
Source: Cancers (Basel). 2025 Apr 30;17(9):1532. doi: 10.3390/cancers17091532 (PMC12071036; doi:10.3390/cancers17091532)
Supplement: Supplementary file 1 [file cancers-17-01532-s001.zip › 1. PRISMA_2020_abstract_checklist hemangioma.pdf]

PRISMA 2020 for Abstracts Checklist

Section and Topic	Item #	Checklist item	Reported (Yes/No)
**TITLE**			
Title	1	Cardiac Hemangiomas: A Five-Year Systematic Review of Diagnosis, Treatment, and Outcomes	Yes
**BACKGROUND**			
Objectives	2	The main objective of this systematic review was to provide an updated analysis of cardiac hemangioma cases published between 2019 and 2025, with specific focus on: epidemiology, histopathological subtypes, clinical presentation, diagnostic approaches, treatment strategies, follow-up outcomes.<br>This review aimed to enhance understanding of the clinical behavior of cardiac hemangiomas and identify areas in need of standardized diagnostic and management protocols.	Yes
**METHODS**			
Eligibility criteria	3	Inclusion Criteria:<br>• Publication Type: Case reports or case series<br>• Time Frame: Published between January 2019 and March 2025<br>• Focus: Specifically addressed cardiac hemangiomas or hemangiomas of the heart<br>• Language: Published in English<br>Exclusion Criteria:<br>• Topic Mismatch: Focused on other vascular tumors (e.g., hemangioendotheliomas, angiosarcomas) or non-cardiac hemangiomas<br>• Study Type: literature reviews, editorials, letters, conference abstracts without original patient data<br>• Duplicate Reports: Re-publication of previously reported cases<br>• Non-English Language: Articles not published in English	Yes
Information sources	4	A systematic literature search was conducted using the PubMed database to identify relevant studies on cardiac hemangiomas. Search coverage period: January 1, 2019 – March 1, 2025. Last date searched: March 1, 2025<br>No additional databases, trial registries, or grey literature sources were searched. Only peer-reviewed, English-language case reports and case series were considered for inclusion.	Yes
Risk of bias	5	No risk of bias was detailed in the abstract.	No
Synthesis of results	6	No methods were detailed to present and synthesise results in the abstract.	No
**RESULTS**			
Included studies	7	A total of 54 studies were included in the review, comprising of 55 individual cases of cardiac hemangiomas published between 2019 and 2025. All included studies were either case reports or small case series, each describing a unique patient.	Yes

PRISMA 2020 for Abstracts Checklist

Section and Topic	Item #	Checklist item	Reported (Yes/No)
Synthesis of results	8	A total of 55 cases were analyzed.<br>• Most common subtype: Cavernous hemangiomas<br>• Age range: From infancy to over 85 years<br>• Sex distribution: Slight predominance in females<br>• Presentation: Ranged from asymptomatic to symptoms like dyspnea and chest pain<br>• Diagnosis: Echocardiography used in over 80% of cases<br>• Treatment: Surgical resection performed in 87.3%<br>• Outcomes: Favorable results with low recurrence, though follow-up was inconsistent and long-term outcomes underreported	Yes
**DISCUSSION**			
Limitations of evidence	9	The evidence included in this review is limited by several factors:<br>• Inconsistency in follow-up: Many cases lacked long-term monitoring, making it difficult to assess recurrence and outcomes over time.<br>• Underreporting of outcomes: Long-term results were often not documented, reducing confidence in treatment durability.<br>• Potential reporting bias: As the review is based on case reports and series, there is a risk of selective reporting and lack of standardization across studies.<br>• Imprecision: Small sample sizes and variability in case detail contribute to uncertainty in the findings.	No
Interpretation	10	This review highlights that while cardiac hemangiomas are rare, the number of reported cases has increased in recent years—likely due to advances in diagnostic imaging and improved clinical awareness. The findings confirm that surgical resection remains the preferred treatment, offering favorable outcomes with low recurrence.<br>However, the review also reveals important gaps: inconsistent follow-up and underreporting of long-term outcomes limit the ability to fully understand prognosis and recurrence risk. These limitations underscore the need for standardized diagnostic and reporting protocols and support the call for further studies to refine classification systems and establish evidence-based guidelines for managing this rare tumor type.	No
**OTHER**			
Funding	11	The primary source of funding for this review was Victor Babes University of Medicine and Pharmacy Timișoara, which supported publication-related costs.	Yes
Registration	12	N/A	No
