# Supplementary material for: Cardiac Hemangiomas: A Five-Year Systematic Review of Diagnosis, Treatment, and Outcomes
Source: Cancers (Basel). 2025 Apr 30;17(9):1532. doi: 10.3390/cancers17091532 (PMC12071036; doi:10.3390/cancers17091532)
Supplement: Supplementary file 1 [file cancers-17-01532-s001.zip › 2. PRISMA_2020_checklist hemangioma.pdf]

PRISMA 2020 Checklist

Section and Topic	Item #	Checklist item	Location where item is reported (page no.)
**TITLE**			
Title	1	**Cardiac Hemangiomas: A Five-Year Systematic Review of Diagnosis, Treatment, and Outcomes**	1
**ABSTRACT**			
Abstract	2	See the attached document (Prisma 2020 for Abstract checklist)	1-2
**INTRODUCTION**			
Rationale	3	Cardiac hemangiomas represent a rare subset of primary cardiac tumors. Over the past five years, significant advancements have been made in the diagnosis and management of cardiac hemangiomas.	3
Objectives	4	This review aims to analyze recent literature on cardiac hemangiomas, focusing on epidemiology, clinical presentation, imaging characteristics, therapeutic strategies, and patient outcomes. By synthesizing the most recent data, we seek to provide a comprehensive update on the current understanding of cardiac hemangiomas and highlight emerging trends that may shape future research in this field	3
**METHODS**			
Eligibility criteria	5	Inclusion criteria: cases of from 2019–2025 focused on 'cardiac hemangioma'; exclusion of reviews and duplicates and cardiac hemangioendothelioma.	3 - 4
Information sources	6	Sources: PubMed; search date: March 1, 2025.	3 - 4
Search strategy	7	A search was conducted in the PubMed database on March 1, 2025, focusing on cardiac hemangiomas and utilizing filters for articles published in the last five years. This initial search returned 233 results. After applying inclusion criteria to select only studies explicitly addressing both 'cardiac hemangioma' and 'hemangioma of the heart', 75 articles remained. Subsequently, an additional manual screening was performed to exclude irrelevant records, such as hemangioendotheliomas, literature reviews, and duplicate case reports, resulting in the removal of 21 more articles.	3 - 4
Selection process	8	The study selection process was conducted by a team of six reviewers (I.R.M., R.C.N., A.P.M., C.N.D., L.S.F., and A.R.M.), who screened the records in pairs of two, ensuring that the entire database was reviewed independently three times. Each pair applied the inclusion criteria to identify studies explicitly addressing both "cardiac hemangioma" and "hemangioma of the heart." Following this, the independently gathered data were combined, cross-verified, and confronted for accuracy. Any discrepancies were discussed and resolved collaboratively. The entire selection process was supervised by C.G.S., and the final approval of the included studies was provided by H.B.F.<br>No automation tools were used in the selection process. Manual screening was employed to exclude irrelevant articles such as hemangioendotheliomas, literature reviews, and duplicate case reports, resulting in a final dataset of 55 eligible cases for analysis.	3 - 4,<br>Appendix A (16-20)
Data collection process	9	Data extraction was carried out by the same team of six reviewers (I.R.M., R.C.N., A.P.M., C.N.D., L.S.F., and A.R.M.), working in pairs of two to ensure consistency and minimize bias. Each pair independently reviewed the assigned set of studies and extracted key information including patient demographics, tumor characteristics, imaging modalities, clinical presentation, treatment strategies, and follow-up outcomes. Following independent extraction, the data were pooled, compared, and cross-validated by all pairs to resolve any discrepancies.<br>The final compiled dataset was reviewed for completeness and consistency under the supervision of C.G.S., and validated by the senior author H.B.F. No automation tools were used in the data collection process, and no additional data were obtained from original study investigators, as all included studies were published case reports or series with publicly available information.	3 - 4, Appendix A (16-20)
Data items	10a	The following data items were collected:<br>• Demographic variables: age, sex	3 - 4, Appendix A (16-20)

Section and Topic	Item #	Checklist item	Location where item is reported (page no.)
		• Tumor characteristics: histological subtype (e.g., cavernous, capillary, mixed, arteriovenous), anatomical location (e.g., right atrium, ventricle, valves), and tumor size (in cm)<br>• Clinical presentation: symptoms at diagnosis (e.g., dyspnea, chest pain, palpitations, asymptomatic)<br>• Diagnostic modalities: primary and secondary imaging techniques (e.g., echocardiography, CT, X-ray)<br>• Treatment strategies: type of intervention (surgical, non-surgical, biopsy-only)<br>• Follow-up data: duration of post-treatment monitoring (in months), and recurrence status (e.g., no recurrence, stable disease, reduced tumor size)<br>All results that were compatible with each outcome domain were sought for each case, regardless of variations in how the data were originally reported. When multiple time points or repeated measures were present, the most complete and clearly defined data points were prioritized, and, in the absence of consistent follow-up timelines, data from the last reported follow-up visit were extracted. No imputation of missing values or estimation techniques were applied.	
	10b	Additional Data Items and Assumptions:<br>In addition to primary clinical and pathological outcomes, the review collected the following secondary variables to enhance contextual analysis:<br>• Co-occurring conditions or tumors: including presence of other benign or malignant tumors (e.g., liver hemangiomas, colon or endometrial cancer) and hematologic disorders<br>• Surgical and procedural details: such as biopsy-only procedures or presence of complications (e.g., postoperative atrioventricular block)<br>• Institutional or study-level details: geographic distribution of reports (when available), journal or source type<br>When missing or unclear information was encountered (e.g., unspecified tumor size, undetermined follow-up duration, or recurrence status), these fields were marked as “not specified” (ns) and excluded from statistical analysis. No assumptions or estimations were made to compensate for these omissions. All included data were extracted as reported in the original publications without direct author contact.	3 - 4, Appendix A (16-20)
Study risk of bias assessment	11	Given that this systematic review included only case reports and case series, a formal risk of bias assessment tool was not applied. These study types inherently present limitations such as lack of control groups, selection bias, reporting variability, and limited generalizability, which were acknowledged during data interpretation.<br>Nevertheless, to ensure data reliability, all included reports were independently reviewed by pairs of reviewers (from among I.R.M., R.C.N., A.P.M., C.N.D., L.S.F., and A.R.M.) for clarity, completeness, and consistency of reported outcomes. Discrepancies or uncertainties were discussed among the review team and resolved by consensus. The process was supervised by C.G.S., with final review oversight by H.B.F.<br>No automated tools were used in the risk of bias evaluation. The review team adhered to standardized criteria to minimize subjectivity in interpreting the findings.	3 - 4, Appendix A (16-20)
Effect measures	12	Effect Measures: given the descriptive nature of this systematic review and the reliance on case reports and case series, no comparative effect measures such as risk ratios, odds ratios, or mean differences were calculated. Instead, the synthesis of results was conducted using descriptive statistics, including:<br>• Frequencies and percentages for categorical variables (e.g., tumor subtypes, anatomical distribution, treatment modalities, recurrence patterns)<br>• Means, medians, ranges, interquartile ranges, and standard deviations for continuous variables (e.g., patient age, tumor size, follow-up duration)	3 - 4, Appendix A (16-20)

PRISMA 2020 Checklist

Section and Topic	Item #	Checklist item	Location where item is reported (page no.)
		These measures were used to summarize trends and characteristics across the 55 included cases. No inferential statistics or modeling techniques were employed due to the heterogeneity and small sample size of individual reports.	
Synthesis methods	13a	Synthesis Methods:<br>All 55 studies included in the final analysis were deemed eligible for synthesis based on predefined inclusion criteria, which required that reports focus explicitly on *cardiac hemangiomas* and provide extractable clinical data. No grouping or exclusion was made based on treatment type or outcome, as the goal of the synthesis was descriptive rather than comparative.<br>The synthesis was conducted through the tabulation of relevant variables, including tumor subtype, anatomical location, patient demographics, clinical presentation, imaging modalities, treatment approach, and follow-up outcomes. Data from each case were standardized and compiled into summary tables and descriptive statistics to facilitate aggregation and interpretation.<br>Visual representations (e.g., histograms, distribution charts etc.) were also employed to illustrate trends such as subtype prevalence, gender distribution, tumor location, and recurrence patterns. No meta-analytic methods were applied due to the heterogeneity and nature of the included studies.	3 - 4, Appendix A (16-20)
	13b	Data Preparation for Synthesis:<br>Before synthesis, all extracted data were standardized and organized into structured tables for descriptive analysis. Continuous variables such as age, tumor size, and follow-up duration were converted into consistent numerical formats (e.g., centimeters for tumor size, months for follow-up) to allow for summary statistics such as mean, median, standard deviation, and interquartile range.<br>Categorical variables (e.g., tumor subtype, anatomical site, diagnostic modality) were uniformly coded to ensure consistency across reports. Cases with missing or unclear data were labeled as “not specified (ns),” and were excluded from numerical analysis but retained in the overall qualitative synthesis.<br>No imputation or estimation techniques were used for missing values. Data conversions were limited to unit standardization, and no transformations or re-calculations of reported outcomes were necessary.	3 - 4, Appendix A (16-20)
	13c	Tabulation and Visualization of Results:<br>To facilitate interpretation, the results of individual studies were systematically tabulated using a structured extraction matrix, which included key variables such as age, sex, tumor subtype, anatomical location, clinical presentation, imaging modality, treatment approach, follow-up duration, and recurrence status. These tables served as the foundation for statistical analysis and synthesis.<br>In addition to tabular representation, several visualizations were generated to highlight patterns and trends across cases. These included:<br>• Histograms of tumor subtype distribution (e.g., cavernous vs. capillary)<br>• Bar charts and pie charts for gender distribution, anatomical site frequency, and clinical symptoms<br>• Time-series graphs to show the trend in publication/reporting years (2019–2025)<br>• Boxplots and scatter plots to visualize tumor size variability, age distribution, and follow-up durations<br>These visual aids were used to supplement narrative synthesis and provide clear, accessible summaries of complex datasets.	3 - 11 Appendix A (16-20)
	13d	Synthesis of Results and Rationale:<br>Given the nature of the included studies—comprised entirely of case reports and case series—a meta-analysis was not conducted. The clinical and methodological heterogeneity of the reports, particularly the lack of standardized outcome measures, comparator groups, and sample size variability, rendered quantitative synthesis inappropriate.<br>Instead, the results were synthesized using narrative and descriptive statistical methods. This approach was chosen to ensure the accurate aggregation of real-world data while preserving the diversity of clinical scenarios. Summary measures such as frequencies, percentages, means,	3 - 11 Appendix A (16-20)

Section and Topic	Item #	Checklist item	Location where item is reported (page no.)
		medians, ranges, and standard deviations were calculated for each variable of interest, including tumor subtypes, anatomical locations, age distributions, clinical presentations, treatment strategies, and follow-up outcomes.<br>Visual and tabular synthesis (as previously described) complemented the narrative analysis. No statistical models, heterogeneity tests, or specialized software packages for meta-analysis were applied, as the primary objective was to map clinical patterns rather than compare outcomes statistically.<br>This synthesis method was deemed most appropriate for the dataset, allowing for comprehensive and meaningful interpretation of rare cardiac hemangioma cases without compromising scientific rigor.	
	13e	Exploration of Heterogeneity:<br>Due to the descriptive nature of the review and the reliance on individual case reports and series, no formal statistical tests for heterogeneity were conducted, and meta-regression or subgroup analyses were not applicable. However, qualitative exploration of heterogeneity was performed by stratifying results based on key clinical and pathological variables, including:<br>• Tumor subtype (cavernous, capillary, mixed, arteriovenous, not specified)<br>• Anatomical location within the heart<br>• Patient demographics (age and gender)<br>• Symptomatology and clinical presentation<br>• Treatment modality (surgical vs. non-surgical)<br>• Follow-up duration and recurrence patterns<br>Descriptive comparisons across these strata were used to identify potential patterns and clinical associations, such as the predominance of cavernous hemangiomas or the frequency of surgical treatment in symptomatic cases. These explorations were narrative and visual rather than inferential, given the limited sample size and variability in data reporting.	3 - 11<br>Appendix A<br>(16-20)
	13f	Sensitivity Analyses:<br>No formal sensitivity analyses were conducted in this review due to the descriptive and non-comparative nature of the included studies. The dataset consisted entirely of individual case reports and small series, which inherently limited the ability to apply statistical methods to assess the robustness of synthesized results.<br>However, as a means of internal validation, descriptive patterns and outcomes were cross-checked against subgroups—for example, evaluating consistency in recurrence rates across different tumor subtypes and treatment modalities. In addition, results were re-examined after excluding cases with incomplete follow-up or unspecified recurrence status to confirm that observed trends (e.g., low recurrence after surgery) remained qualitatively consistent.<br>These informal checks supported the stability of key findings, but they do not constitute formal sensitivity testing in the statistical sense.	3 - 11<br>Appendix A<br>(16-20)
Reporting bias assessment	14	Assessment of Risk of Bias Due to Missing Results (Reporting Bias):<br>Due to the inclusion of only case reports and case series, no formal methods were applied to assess risk of bias due to missing results or publication bias. These types of studies are inherently subject to reporting biases, including selective publication of unusual, positive, or surgically treated cases.<br>To partially address this concern, efforts were made to comprehensively search the literature, including both successful and non-surgical outcomes, and to include all cases that met the predefined inclusion criteria, regardless of outcome. The review also documented instances where key data were not reported or were labeled as “not specified (ns)”, and these omissions were noted transparently in tables and excluded from quantitative summaries.	3 - 11<br>Appendix A<br>(16-20)

Section and Topic	Item #	Checklist item	Location where item is reported (page no.)
		Although no statistical assessment of reporting bias was feasible, the potential for underreporting of negative or uneventful cases was acknowledged as a limitation in interpreting the frequency of outcomes such as recurrence, complications, or follow-up duration.	
Certainty assessment	15	Assessment of Certainty in the Body of Evidence:<br>No formal framework, such as the GRADE approach (Grading of Recommendations Assessment, Development and Evaluation), was applied to assess the certainty or confidence in the body of evidence for the reported outcomes, as the review is based solely on individual case reports and case series. These types of studies are inherently associated with low levels of evidence due to lack of control groups, randomization, or standardized outcome measures.<br>Nonetheless, the review attempted to improve confidence in the descriptive findings by:<br>• Applying strict inclusion and exclusion criteria<br>• Ensuring multiple reviewers independently extracted and verified data<br>• Conducting a comprehensive synthesis of clinically and pathologically relevant variables<br>• Highlighting limitations in data completeness and follow-up consistency<br>While the descriptive trends observed (e.g., predominance of cavernous subtypes, low recurrence post-surgery) are consistent with existing literature, the overall certainty in these findings remains limited, and results should be interpreted with appropriate caution.	3 - 11<br>Appendix A (16-20)
**RESULTS**			
Study selection	16a	Study Selection:<br>The initial search of the PubMed database, conducted on March 1, 2025, identified 233 records related to cardiac hemangiomas published in the past five years. After applying inclusion criteria—requiring explicit reference to “cardiac hemangioma” or “hemangioma of the heart”—the pool was narrowed to 75 potentially eligible studies.<br>A subsequent manual screening process was performed to exclude irrelevant records, such as reports on hemangioendotheliomas, literature reviews, and duplicate case reports. This resulted in the exclusion of 21 additional studies, yielding a final total of 54 publications comprising 55 individual cases, all of which met the predefined inclusion criteria and were included in the synthesis.<br>While a PRISMA flow diagram is recommended to visualize this process, the review presented the selection process narratively and supplemented it with a table of included studies (Appendix A).	4 - 11<br>Appendix A (16-20)
	16b	The following studies were excluded because the location of the tumor was extracardiac:<br>• D. Jacob, T. Pratap, A. Kumar, R. Rashmi, and A. K. Vishnu, “Benign Pericardial Hemangioma-A Rare Cause of Cardiac Tamponade,” *Indian Journal of Radiology and Imaging*, vol. 31, no. 3, pp. 754–757, Jul. 2021, doi: 10.1055/s-0041-1736405.<br>• N. Kalinic *et al.*, “Pericardial hemangioma: An extremely rare cardiac tumor,” *Kardiol Pol*, vol. 82, no. 1, pp. 105–106, 2024, doi: 10.33963/v.kp.98422.<br>• F. Adomat, D. A. Steffen, L. Suter-Magpantay, A. Linka, and L. Weber, “Case report: a non-invasive approach to diagnosis and management of pericardial haemangioma,” *Eur Heart J Case Rep*, vol. 8, no. 10, Oct. 2024, doi: 10.1093/ehjcr/ytae545.<br>• N. Theerasuwipakorn, S. Peenakhon, P. Varachotisate, W. Srinu, and W. Promratpan, “Giant Pericardial Hemangioma With a Large Central Fibrotic Core,” *CJC Open*, vol. 6, no. 8, pp. 1028–1031, Aug. 2024, doi: 10.1016/j.cjco.2024.05.009.<br>• A. Mitsuishi, Y. Miura, S. Hosogi, M. Tsutsui, and H. Kitaoka, “3 Lobes of Extracardiac Hemangioma,” *JACC Case Rep*, vol. 29, no. 15, Aug. 2024, doi: 10.1016/j.jaccas.2024.102406.<br>• F. Sadegh Beigee, A. Sheikhy, and K. Sheikhy, “Reconstruction of Chest Wall by Cryopreserved Sternum Allograft After Resection of Sternal Hemangioma: A Case Report,” *Front Surg*, vol. 9, Mar. 2022, doi: 10.3389/fsurg.2022.796806.	4 - 11<br>Appendix A (16-20),<br>20-25

Section and Topic	Item #	Checklist item	Location where item is reported (page no.)
		• M. Delaney, K. Hopkins, M. Chugh, R. Murthy, and N. Choueiter, "Multimodality Approach to a Neonate With a Pericardial Mass and a Hemorrhagic Pericardial Effusion," *JACC Case Rep*, vol. 29, no. 24, Dec. 2024, doi: 10.1016/j.jaccas.2024.102921.<br>• F. Barros Alves *et al.*, "Pericardial hemangioma – Imaging with pathologic correlation of an extremely rare mediastinal lesion," *Revista Portuguesa de Cardiologia*, vol. 43, no. 1, pp. 49–50, Jan. 2024, doi: 10.1016/j.repc.2023.02.013.<br>• M. Xu, F. Xiong, L. Zhang, and S. Wang, "Hemangioma mimicking left atrial mass in the posterior mediastinum: A case report with literature review," *Int Heart J*, vol. 62, no. 2, pp. 453–457, 2021, doi: 10.1536/ihj.20-547.<br><br>The following study was excluded because it was a duplicate study:<br>• M. Alsaloum *et al.*, "A Right Atrial Mass Discovered Postpartum: A Diagnostic Challenge," *CASE*, vol. 7, no. 8, pp. 325–330, Aug. 2023, doi: 10.1016/j.case.2023.04.006.<br><br>The following study were excluded because they were diagnosed as hemangioendothelioma:<br>• E. Karaağaç, N. Yeşilkaya, T. M. Tellioğlu, F. Ç. Ünay, and Y. Beşir, "A rare cardiac tumor presenting with myxoma: Primary cardiac hemangioendothelioma," *Turkish Journal of Thoracic and Cardiovascular Surgery*, vol. 29, no. 1, pp. 110–113, 2021, doi: 10.5606/tgkdc.dergisi.2021.20695.<br>• T. Schaeffer, K. Glatz, F. S. Eckstein, and P. Matt, "Composite haemangioendothelioma in the heart: a case report," *Eur Heart J Case Rep*, vol. 7, no. 8, Aug. 2023, doi: 10.1093/ehjcr/ytad343.<br>• P. Langguth, M. S. Ravesh, A. Haneya, and M. Both, "Composite hemangioendothelioma: The first case of a right atrioventricular pericardial tumour," Jun. 01, 2020, *Oxford University Press*. doi: 10.1093/ehjcr/ytaa110.<br>• H. T. Stahel, M. Haranal, and L. Song, "Case report: Right ventricular outflow tract obstruction caused by multicomponent mesenchymal tumor."<br>• W. Huang, L. Li, J. Gao, and J. B. Gao, "Epithelioid hemangioendothelioma of the right atrium invaded the superior vena cava: case report and review of literature," *International Journal of Cardiovascular Imaging*, vol. 37, no. 1, pp. 285–290, Jan. 2021, doi: 10.1007/s10554-020-01963-w.<br>• Y. N. Jin, J. L. Cheng, Y. Zhang, X. N. Shao, X. P. Zhang, and W. B. Zhang, "An mri image analysis of primary cardiac neoplasms," *Int J Gen Med*, vol. 14, pp. 2943–2951, 2021, doi: 10.2147/IJGM.S296381.<br><br>The following study were excluded because they were not case reports:<br>• E. Qiao, Y. Wang, Z. Huang, F. Li, and W. Wang, "Long-term follow-up of resection of primary benign right ventricular tumours: A 10-year surgical experience," *Ann R Coll Surg Engl*, vol. 103, no. 1, pp. 53–58, Jan. 2021, doi: 10.1308/RCSANN.2020.0200.<br>• H. Singh, V. Dasagrandhi, R. Kumar, R. Kumar, and B. R. Mittal, "Impact of 18F-fluorodeoxyglucose positron emission tomography computed tomography imaging in a case of pericardial cavernous hemangioma," *Indian Journal of Nuclear Medicine*, vol. 35, no. 4, pp. 360–361, Oct. 2020, doi: 10.4103/ijnm.IJNM_69_20.<br>• P. Ciliberti *et al.*, "Additional value of cardiac magnetic resonance parametric mapping in tissue characterization of common benign paediatric cardiac tumours," *Eur Heart J Cardiovasc Imaging*, Dec. 2024, doi: 10.1093/ehjci/jeae187.<br>• V. Braun *et al.*, "Congenital haemangiomas: A single-centre retrospective review," Dec. 07, 2020, *BMJ Publishing Group*. doi: 10.1136/bmjpo-2020-000816.	

Section and Topic	Item #	Checklist item	Location where item is reported (page no.)
Study characteristics	17	The following studies were included in our systematic review because they were case reports of cardiac hemangiomas, and we devised them in this fashion:<br>**Cavernous hemangioma:**<br>• F. Qamar *et al.*, "Cardiac Cavernous Hemangioma," *JACC Case Rep*, vol. 30, no. 4, Feb. 2025, doi: 10.1016/j.jaccas.2024.102956.<br>• Y. Kondo *et al.*, "Giant cardiac hemangioma in the right atrium: an asymptomatic surgical case," *General Thoracic and Cardiovascular Surgery Cases*, vol. 2, no. 1, Jul. 2023, doi: 10.1186/s44215-023-00060-3.<br>• T. Kobayashi, S. Numata, Y. Hohri, H. Kawajiri, and H. Yaku, "Cardiac cavernous hemangioma with high fluorodeoxyglucose uptake on preoperative positron emission tomography/computed tomography: a case report," *General Thoracic and Cardiovascular Surgery Cases*, vol. 2, no. 1, Jul. 2023, doi: 10.1186/s44215-023-00054-1.<br>• T. Xie *et al.*, "Rheumatism as a cause of cardiac hemangioma: a rare case report and review of literature with special focus on etiology," *BMC Cardiovasc Disord*, vol. 23, no. 1, Dec. 2023, doi: 10.1186/s12872-023-03241-8.<br>• L. Berdica, E. Kola, D. Nakuci, E. Horjeti, and M. Alimehmeti, "Cardiac hemangioma presenting as a primary cardiac tumor," *Cardio-Oncology*, vol. 9, no. 1, Dec. 2023, doi: 10.1186/s40959-023-00154-5.<br>• P. Wang, D. Chapman, and F. Siddiqui, "A Rare Cardiac Cavernous Hemangioma Treated with Radiotherapy," *Case Rep Vasc Med*, vol. 2022, pp. 1–4, Sep. 2022, doi: 10.1155/2022/5698475.<br>• M. Shashikanth, S. Nicola, C. Yi, and S. Julian, "Right atrial cavernous hemangioma," *Ann Card Anaesth*, vol. 23, no. 3, pp. 335–337, Jul. 2020, doi: 10.4103/aca.ACA_58_19.<br>• J. Wang, D. Li, P. Hu, X. J. Ma, and J. Xie, "Rare Gourd-Shaped Cardiac Hemangioma: Computed Tomography Imaging Characteristics and Clinical Management," *Anatol J Cardiol*, vol. 29, no. 1, pp. E3–E4, Jan. 2025, doi: 10.14744/AnatolJCardiol.2024.4860.<br>• M. H. Anbardar, N. Soleimani, and S. Mohammadzadeh, "Two cases of cardiac hemangioma in different anatomical locations presenting with chest pain and palpitation," *Clin Case Rep*, vol. 10, no. 2, Feb. 2022, doi: 10.1002/ccr3.5495.<br>• R. Thilak, A. Sivanesan, H. Munuswamy, and P. C. Toi, "A Giant Right Atrial Hemangioma- Case Report," *Cureus*, Apr. 2022, doi: 10.7759/cureus.24622.<br>• C. Cattapan *et al.*, "Preoperative Transcatheter Diagnosis of Right Atrial Hemangioma," *JACC Case Rep*, vol. 15, Jun. 2023, doi: 10.1016/j.jaccas.2023.101857.<br>• Y. Fu, H. Ma, and Y. Guo, "Giant cavernous hemangioma in the aortic root and right atrioventricular groove," May 01, 2020, *Mosby Inc*. doi: 10.1016/j.jtcvs.2019.04.020.<br>• M. Toscano, A. R. Alves, C. Matias, M. Carvalho, and M. Marques, "Hemangioma of the mitral valve: Following the murmur," *Revista Portuguesa de Cardiologia*, vol. 41, no. 9, pp. 795–799, Sep. 2022, doi: 10.1016/j.repc.2022.07.003.<br>• E. B. Kesieme and K. G. Buchan, "Multiple Right Ventricular Haemangiomas," *Cureus*, Mar. 2023, doi: 10.7759/cureus.36570.<br>• T. T. Vu *et al.*, "A case of a small-sized cavernous hemangioma in the right ventricle - an incidental finding," *Radiol Case Rep*, vol. 17, no. 3, pp. 856–862, Mar. 2022, doi: 10.1016/j.radcr.2021.12.038.<br>• F. Dobritoiu, H. Moldovan, R. Oncica, G. Vasile, E. Nechifor, and C. Copaescu, "Giant cavernous hemangioma of the right atrium – A rare case and literature review," Apr. 01, 2020, *Editura Celsius*. doi: 10.21614/chirurgia.115.2.267.<br>• G. Drevet, L. Chalabreysse, D. Gamondes, F. Tronc, and J. M. Maury, "Epicardial carvernous hemangioma: The diagnostic challenge	4 - 11<br>Appendix A (16-20),<br>20-25

Section and Topic	Item #	Checklist item	Location where item is reported (page no.)
		of a middle mediastinal mass," *Thorac Cancer*, vol. 12, no. 17, pp. 2404–2406, Sep. 2021, doi: 10.1111/1759-7714.14074.<br>• O. Parkash, G. W. Ying, A. Ram, L. P. Vemireddy, and F. Zahra, "A Rare Case of Cavernous Hemangioma of the Mitral Valve Presenting As Multifocal Embolic Brain Infarcts," *Cureus*, Sep. 2021, doi: 10.7759/cureus.17721.<br>• L. Ku, Y. Chen, Y. Wang, Z. Liu, and X. Ma, "IMAGE OF THE MONTH Multimodality imaging for the diagnosis of giant cavernous hemangioma of the right ventricle", doi: 10.1016/j.hjc.2024.1.<br>• N. Bayfield, L. Bibo, E. Wang, and J. Passage, "Cavernous haemangioma of anterior mitral valve leaflet: Diagnostic utility of 3D echocardiography," *BMJ Case Rep*, vol. 15, no. 2, Feb. 2022, doi: 10.1136/bcr-2021-247352.<br>• M. Tang, Z. Jian, Y. Yan, and F. Guo, "Right ventricular haemangioma as a rare cause of chest pain: A case report," *Eur Heart J Case Rep*, vol. 5, no. 12, Dec. 2021, doi: 10.1093/ehjcr/ytab477.<br>• R. Cheaban, M. Piran, D. Opacic, J. F. Gummert, and S. V. Rojas, "Epicardial cavernous haemangioma; A case report of a unique incidental finding," *Eur Heart J Case Rep*, vol. 8, no. 4, Apr. 2024, doi: 10.1093/ehjcr/ytae146.<br>• S. Harrison, "Rare Case of Cavernous Haemangioma of the Right Atrium with Probable Hepatic Haemangioma," *Case Rep Cardiol*, vol. 2022, pp. 1–4, Feb. 2022, doi: 10.1155/2022/9214196.<br>**Capillary hemangioma:**<br>• M. H. Anbardar, N. Soleimani, and S. Mohammadzadeh, "Two cases of cardiac hemangioma in different anatomical locations presenting with chest pain and palpitation," *Clin Case Rep*, vol. 10, no. 2, Feb. 2022, doi: 10.1002/ccr3.5495.<br>• H. Osada *et al.*, "Cardiac capillary hemangioma originating from the mitral valve," *JTCVS Tech*, vol. 20, pp. 127–129, Aug. 2023, doi: 10.1016/j.xjtc.2023.05.002.<br>• N. Kalinic *et al.*, "Pericardial hemangioma: An extremely rare cardiac tumor," *Kardiol Pol*, vol. 82, no. 1, pp. 105–106, 2024, doi: 10.33963/v.kp.98422.<br>• T. Nakajima, T. Shibata, K. Ogura, Y. Iba, and N. Kawaharada, "A Case of a Giant Hemangioma of a Primary Cardiac Tumor," *Cureus*, Aug. 2023, doi: 10.7759/cureus.43818.<br>• J. Gourmelon, V. Loobuyck, V. Silvestri, A. Chaput, and A. Altes, "Right Atrial Cardiac Hemangioma: A Multidisciplinary Pathway From Symptoms to Surgery," *JACC Case Rep*, vol. 29, no. 24, Dec. 2024, doi: 10.1016/j.jaccas.2024.102920.<br>• R. Rocco, R. Daly, and A. Arghami, "Robotic-Assisted Resection of Rare Mitral Valve Hemangioma," *Mayo Clin Proc Innov Qual Outcomes*, vol. 8, no. 3, pp. 249–252, Jun. 2024, doi: 10.1016/j.mayocpiqo.2024.03.007.<br>• H. C. Nguyen and D. T. Pham, "Totally endoscopic resection of epicardial cardiac haemangioma under on-pump beating heart," *Annals of Medicine and Surgery*, vol. 69, Sep. 2021, doi: 10.1016/j.amsu.2021.102838.<br>• E. Moreno-Pallares, D. Vargas-Vergara, A. Bornacelly, J. Gutiérrez, A. Olaya-Sánchez, and M. Velasco-Morales, "Right ventricular capillary hemangioma as a cause of congestive heart failure: case report and review of the literature," Apr. 01, 2024, *Instituto Nacional de Cardiologia Ignazio Chavez*. doi: 10.24875/ACM.23000097.<br>• S. Yildirim, M. Işik, Ö. Tanyeli, and N. Görmüş, "Giant left atrial capillary haemangioma invading left-main coronary artery," *Interact Cardiovasc Thorac Surg*, vol. 33, no. 4, pp. 631–633, Oct. 2021, doi: 10.1093/icvts/ivab133.<br>• Darbari, D. Singh, S. Gilbert, B. Kumar, and N. Singh, "Capillary haemangioma of the heart presenting with pericardial effusion: A case report," *J Cardiovasc Thorac Res*, vol. 13, no. 3, pp. 250–253, Aug. 2021, doi: 10.34172/jcvtr.2020.60.<br>**Cavernous-capillary hemangioma:**<br>• J. Y. Campos, D. R. da Silva, A. P. T. Cardoso, N. A. G. Stolf, and G. Pozzan, "Cardiac papillary muscle hemangioma," *Autops Case*	

Section and Topic	Item #	Checklist item	Location where item is reported (page no.)
		Rep, vol. 10, no. 2, 2020, doi: 10.4322/acr.2020.169.<br>• C. M. Yang and Y. N. Hu, "Cardiac Hemangioma Mimicking Infective Endocarditis," *Diagnostics*, vol. 14, no. 19, Oct. 2024, doi: 10.3390/diagnostics14192109.<br>• F. Rattenni *et al.*, "Advanced presentation of cardiac hemangioma," *J Cardiothorac Surg*, vol. 19, no. 1, p. 620, Dec. 2024, doi: 10.1186/s13019-024-02984-5.<br>• D. Caicedo, K. Oshiro, J. S. Glickstein, U. Krishnan, and M. P. DiLorenzo, "Cardiac Hemangioma in an Asymptomatic Teenager with a History of Congenital Heart Disease," *CASE*, vol. 4, no. 5, pp. 362–364, Oct. 2020, doi: 10.1016/j.case.2020.06.005.<br>• N. Kaewboonlert *et al.*, "Right ventricular outflow tract obstruction by cardiac hemangioma in asymptomatic patient," *J Surg Case Rep*, vol. 2024, no. 5, May 2024, doi: 10.1093/jscr/rjae321.<br>• S. Takago, K. Iino, Y. Yamamoto, and H. Takemura, "Cardiac haemangioma treated with surgical resection involving reconstruction of the right ventricle," *Interact Cardiovasc Thorac Surg*, vol. 32, no. 1, pp. 153–155, Jan. 2021, doi: 10.1093/icvts/ivaa216.<br>• Y. Miyoshi, T. Kitai, T. Yamane, M. Sano, T. Koyama, and Y. Furukawa, "A huge cardiac haemangioma in the left ventricular wall," Dec. 01, 2020, *Oxford University Press*. doi: 10.1093/ehjcr/ytaa374.<br>• D. G. Abdul-Rahman *et al.*, "Patient with myelodysplastic syndrome presented with recurrent pericardial effusion diagnosed as epicardial hemangioma; Case report of a rare diagnosis with rare presentation," *Radiol Case Rep*, vol. 18, no. 6, pp. 2253–2258, Jun. 2023, doi: 10.1016/j.radcr.2023.03.028.<br><br>**Arterio-venous hemangioma:**<br>• M. Piao, X. Zhou, and M. Yan, "Exploring the Causes of Newly Developed Mitral Valve Regurgitation after the Resection of a Giant Left Ventricular Tumor (Hemangioma)," *J Cardiothorac Vasc Anesth*, Mar. 2024, doi: 10.1053/j.jvca.2024.10.042.<br>• H. Liu, X. Li, C. Zhang, C. Fan, L. Liu, and J. Wan, "Case Report: A Primary Right Ventricular Vascular Malformation Presenting as a Mass," *Front Cardiovasc Med*, vol. 8, 2021, doi: 10.3389/fcvm.2021.736199.<br>**Not specified hemangioma:**<br>• M. Shah, L. P. Russo, D. Haddad, J. Chang, and A. Okere, "Cardiac Hemangioma: A Rare Tumor Presenting as Postpartum Chest Pain," *Cureus*, Aug. 2023, doi: 10.7759/cureus.44407.<br>• J. Batko, D. J. Rams, K. Bartuś, A. Bartoszcze, and R. A. Litwinowicz, "Cardiac hemangioma in the atrioventricular node localization," 2023, *Termedia Publishing House Ltd.* doi: 10.5114/kitp.2023.126102.<br>• L. Ilcheva, M. Cholubek, D. Loiero, and O. Dzemali, "Cardiac Hemangioma in the Left Ventricular Septum," *Thorac Cardiovasc Surg Rep*, vol. 13, no. 01, pp. e4–e7, Jan. 2024, doi: 10.1055/s-0044-1778719.<br>• J. Fan *et al.*, "Diagnostic mystery—a rare right ventricular cardiac hemangioma: a case report," *J Cardiothorac Surg*, vol. 16, no. 1, Dec. 2021, doi: 10.1186/s13019-021-01731-4.<br>• Y. A. Zonooz *et al.*, "Cardiac interventricular septum hemangioma in a colon cancer patient treated with Capecitabine: A case report and review of literature," *Clin Case Rep*, vol. 12, no. 8, Aug. 2024, doi: 10.1002/ccr3.9331.<br>• F. Liu, M. Dong, and Q. Li, "Lobulated Hemangioma as a Rare Cause of Tricuspid Regurgitation," *Clinical Medicine Insights: Case Reports* , vol. 17, Jan. 2024, doi: 10.1177/11795476241274699.<br>• Dursun, A. Hakgor, M. Z. Kenger, and O. Karaca, "Concomitant Right Atrial Hemangioma Resection With LVAD Implantation: First-in-Human Experience," *JACC Case Rep*, vol. 29, no. 24, Dec. 2024, doi: 10.1016/j.jaccas.2024.102860.	

Section and Topic	Item #	Checklist item	Location where item is reported (page no.)
		• Bernal-Gallego, V. Hernández-Jiménez, L. Castillo, R. González-Davia, N. De Antonio-Antón, and G. Reyes-Copa, "Unexpected diagnosis: large hemangioma in the interatrial septum," *J Cardiothorac Surg*, vol. 19, no. 1, Dec. 2024, doi: 10.1186/s13019-024-02794-9.<br>• R. S. Al Umairi and S. Sabek, "Left ventricle intramuscular haemangioma a case report and review of literature," *Sultan Qaboos Univ Med J*, vol. 21, no. 2, pp. e316–e319, Jun. 2021, doi: 10.18295/squmj.2021.21.02.024.<br>• J. Chen, X. Gong, L. Xie, Q. Wu, T. Zhao, and S. Hu, "Atrial septal defect with a rare occupying lesion in heart," *BMC Cardiovasc Disord*, vol. 22, no. 1, Dec. 2022, doi: 10.1186/s12872-022-02919-9.<br>• Sengupta, G. LaRocca, M. E. Goldman, D. H. Adams, and A. C. Anyanwu, "Hemangioma of the Mitral Valve and Aortomitral Curtain in an Adolescent," *JACC Case Rep*, vol. 29, no. 24, Dec. 2024, doi: 10.1016/j.jaccas.2024.102840.<br>• M. Pereira, C. Almeida, J. Antunes-Sarmento, and J. O. Miranda, "Refractory Fetal and Neonatal Supraventricular Tachycardia Associated With Mitral Valve Mass," *Cureus*, Jul. 2024, doi: 10.7759/cureus.63944.	
Risk of bias in studies	18	Possible risk of bias in the following case studies may arise due to incomplete clinical details such as missing follow-up data and no confirmation of histopathological type:<br>• M. Shah, L. P. Russo, D. Haddad, J. Chang, and A. Okere, "Cardiac Hemangioma: A Rare Tumor Presenting as Postpartum Chest Pain," *Cureus*, Aug. 2023, doi: 10.7759/cureus.44407.<br>• J. Batko, D. J. Rams, K. Bartuś, A. Bartoszcze, and R. A. Litwinowicz, "Cardiac hemangioma in the atrioventricular node localization," 2023, *Termedia Publishing House Ltd.* doi: 10.5114/kitp.2023.126102.<br>• J. Y. Campos, D. R. da Silva, A. P. T. Cardoso, N. A. G. Stolf, and G. Pozzan, "Cardiac papillary muscle hemangioma," *Autops Case Rep*, vol. 10, no. 2, 2020, doi: 10.4322/acr.2020.169.<br>• C. M. Yang and Y. N. Hu, "Cardiac Hemangioma Mimicking Infective Endocarditis," *Diagnostics*, vol. 14, no. 19, Oct. 2024, doi: 10.3390/diagnostics14192109.<br>• L. Berdica, E. Kola, D. Nakuci, E. Horjeti, and M. Alimehmeti, "Cardiac hemangioma presenting as a primary cardiac tumor," *Cardio-Oncology*, vol. 9, no. 1, Dec. 2023, doi: 10.1186/s40959-023-00154-5.<br>• N. Kalinic *et al.*, "Pericardial hemangioma: An extremely rare cardiac tumor," *Kardiol Pol*, vol. 82, no. 1, pp. 105–106, 2024, doi: 10.33963/v.kp.98422.<br>• L. Ilcheva, M. Cholubek, D. Loiero, and O. Dzemali, "Cardiac Hemangioma in the Left Ventricular Septum," *Thorac Cardiovasc Surg Rep*, vol. 13, no. 01, pp. e4–e7, Jan. 2024, doi: 10.1055/s-0044-1778719.<br>• T. Nakajima, T. Shibata, K. Ogura, Y. Iba, and N. Kawaharada, "A Case of a Giant Hemangioma of a Primary Cardiac Tumor," *Cureus*, Aug. 2023, doi: 10.7759/cureus.43818.<br>• H. Liu, X. Li, C. Zhang, C. Fan, L. Liu, and J. Wan, "Case Report: A Primary Right Ventricular Vascular Malformation Presenting as a Mass," *Front Cardiovasc Med*, vol. 8, 2021, doi: 10.3389/fcvm.2021.736199.<br>• M. Shashikanth, S. Nicola, C. Yi, and S. Julian, "Right atrial cavernous hemangioma," *Ann Card Anaesth*, vol. 23, no. 3, pp. 335–337, Jul. 2020, doi: 10.4103/aca.ACA_58_19.<br>• J. Gourmelon, V. Loobuyck, V. Silvestri, A. Chaput, and A. Altes, "Right Atrial Cardiac Hemangioma: A Multidisciplinary Pathway From Symptoms to Surgery," *JACC Case Rep*, vol. 29, no. 24, Dec. 2024, doi: 10.1016/j.jaccas.2024.102920.<br>• Y. A. Zonooz *et al.*, "Cardiac interventricular septum hemangioma in a colon cancer patient treated with Capecitabine: A case report	4 - 11 Appendix A (16-20),<br>20-25

Section and Topic	Item #	Checklist item	Location where item is reported (page no.)
		and review of literature," *Clin Case Rep*, vol. 12, no. 8, Aug. 2024, doi: 10.1002/ccr3.9331.<br>• Y. Fu, H. Ma, and Y. Guo, "Giant cavernous hemangioma in the aortic root and right atrioventricular groove," May 01, 2020, *Mosby Inc*. doi: 10.1016/j.jtcvs.2019.04.020.<br>• F. Liu, M. Dong, and Q. Li, "Lobulated Hemangioma as a Rare Cause of Tricuspid Regurgitation," *Clinical Medicine Insights: Case Reports* , vol. 17, Jan. 2024, doi: 10.1177/11795476241274699.<br>• T. T. Vu *et al.*, "A case of a small-sized cavernous hemangioma in the right ventricle - an incidental finding," *Radiol Case Rep*, vol. 17, no. 3, pp. 856–862, Mar. 2022, doi: 10.1016/j.radcr.2021.12.038.<br>• F. Dobritoiu, H. Moldovan, R. Oncica, G. Vasile, E. Nechifor, and C. Copaescu, "Giant cavernous hemangioma of the right atrium – A rare case and literature review," Apr. 01, 2020, *Editura Celsius*. doi: 10.21614/chirurgia.115.2.267.<br>• Dursun, A. Hakgor, M. Z. Kenger, and O. Karaca, "Concomitant Right Atrial Hemangioma Resection With LVAD Implantation: First-in-Human Experience," *JACC Case Rep*, vol. 29, no. 24, Dec. 2024, doi: 10.1016/j.jaccas.2024.102860.<br>• Bernal-Gallego, V. Hernández-Jiménez, L. Castillo, R. González-Davia, N. De Antonio-Antón, and G. Reyes-Copa, "Unexpected diagnosis: large hemangioma in the interatrial septum," *J Cardiothorac Surg*, vol. 19, no. 1, Dec. 2024, doi: 10.1186/s13019-024-02794-9.<br>• H. C. Nguyen and D. T. Pham, "Totally endoscopic resection of epicardial cardiac haemangioma under on-pump beating heart," *Annals of Medicine and Surgery*, vol. 69, Sep. 2021, doi: 10.1016/j.amsu.2021.102838.<br>• R. S. Al Umairi and S. Sabek, "Left ventricle intramuscular haemangioma a case report and review of literature," *Sultan Qaboos Univ Med J*, vol. 21, no. 2, pp. e316–e319, Jun. 2021, doi: 10.18295/squmj.2021.21.02.024.<br>• O. Parkash, G. W. Ying, A. Ram, L. P. Vemireddy, and F. Zahra, "A Rare Case of Cavernous Hemangioma of the Mitral Valve Presenting As Multifocal Embolic Brain Infarcts," *Cureus*, Sep. 2021, doi: 10.7759/cureus.17721.<br>• L. Ku, Y. Chen, Y. Wang, Z. Liu, and X. Ma, "IMAGE OF THE MONTH Multimodality imaging for the diagnosis of giant cavernous hemangioma of the right ventricle", doi: 10.1016/j.hjc.2024.1.<br>• E. Moreno-Pallares, D. Vargas-Vergara, A. Bornacelly, J. Gutiérrez, A. Olaya-Sánchez, and M. Velasco-Morales, "Right ventricular capillary hemangioma as a cause of congestive heart failure: case report and review of the literature," Apr. 01, 2024, *Instituto Nacional de Cardiologia Ignazio Chavez*. doi: 10.24875/ACM.23000097.<br>• J. Chen, X. Gong, L. Xie, Q. Wu, T. Zhao, and S. Hu, "Atrial septal defect with a rare occupying lesion in heart," *BMC Cardiovasc Disord*, vol. 22, no. 1, Dec. 2022, doi: 10.1186/s12872-022-02919-9.<br>• S. Yildirim, M. Işik, Ö. Tanyeli, and N. Görmüş, "Giant left atrial capillary haemangioma invading left-main coronary artery," *Interact Cardiovasc Thorac Surg*, vol. 33, no. 4, pp. 631–633, Oct. 2021, doi: 10.1093/icvts/ivab133.<br>• N. Bayfield, L. Bibo, E. Wang, and J. Passage, "Cavernous haemangioma of anterior mitral valve leaflet: Diagnostic utility of 3D echocardiography," *BMJ Case Rep*, vol. 15, no. 2, Feb. 2022, doi: 10.1136/bcr-2021-247352.<br>• Sengupta, G. LaRocca, M. E. Goldman, D. H. Adams, and A. C. Anyanwu, "Hemangioma of the Mitral Valve and Aortomitral Curtain in an Adolescent," *JACC Case Rep*, vol. 29, no. 24, Dec. 2024, doi: 10.1016/j.jaccas.2024.102840.<br>• M. Pereira, C. Almeida, J. Antunes-Sarmento, and J. O. Miranda, "Refractory Fetal and Neonatal Supraventricular Tachycardia Associated With Mitral Valve Mass," *Cureus*, Jul. 2024, doi: 10.7759/cureus.63944.	

Section and Topic	Item #	Checklist item	Location where item is reported (page no.)
Results of individual studies	19	Since individual case reports do not contain control groups or statistical comparisons, no effect estimates or confidence intervals are reported. Instead, outcomes were summarized using descriptive statistics per case.	4 - 11 Appendix A (16-20)
Results of syntheses	20a	The synthesis included 55 cases of cardiac hemangiomas extracted from individual case reports and small case series published between 2019 and 2025. Most reports originated from a variety of global settings, including North America, Europe, Asia, and South America, and covered a broad demographic spectrum (ages ranged from under 1 year to 87 years; 58.2% female). The most commonly reported subtype was cavernous, with the right atrium being the most frequently involved anatomical site. Nearly all patients underwent surgical excision, with a minority treated conservatively or with biopsy-only approaches.<br>Given the nature of the evidence base—uncontrolled, descriptive studies with heterogeneous reporting standards—a formal risk of bias assessment was not feasible. However, notable limitations include:<br>• Reporting variability, particularly in tumor measurements, follow-up duration, and recurrence status<br>• Incomplete outcome data in several reports, with missing details labeled as “not specified (ns)”<br>• A likely publication bias favoring successful surgical outcomes and unusual or dramatic presentations<br>• Lack of standardized imaging or diagnostic criteria across cases<br>Despite these constraints, consistency in patterns such as low recurrence post-surgery and frequent use of echocardiography for diagnosis helped support the reliability of the descriptive synthesis.	4 - 11 Appendix A (16-20)
	20b	Statistical Synthesis of Results:<br>No meta-analyses were performed in this review due to the case-based nature of the included studies and the absence of control or comparator groups. Instead, a descriptive statistical synthesis was carried out across all 55 cases to identify trends in clinical, pathological, and management-related outcomes.<br>Descriptive results included:<br>• Frequencies and proportions for categorical variables such as tumor subtype (e.g., cavernous: 40%, capillary: 18%), anatomical location (e.g., right atrium most common), clinical symptoms (e.g., dyspnea, palpitations), and recurrence (e.g., 90% reported no recurrence).<br>• Means, medians, and ranges were reported for continuous variables such as tumor size (e.g., median 3.76 cm), patient age (e.g., mean 52.2 years), and follow-up duration (e.g., median 12 months).<br>No subgroup analyses or effect size estimates were generated. Additionally, no statistical heterogeneity measures were applicable due to the non-comparative, descriptive design. Observed trends suggest favorable outcomes post-surgery and low complication rates, but these should be interpreted with caution given the inherent biases and variability in case report data.	4 - 11 Appendix A (16-20)
	20c	Sources of Heterogeneity in Study Results<br>1. Tumor Histological Subtypes<br>• Cavernous hemangiomas were the most frequently reported (23 cases).<br>• Other subtypes included capillary, cavernous-capillary (mixed), and arteriovenous hemangiomas, with 12 cases marked as “not specified.”<br>• Variation in subtype reporting may stem from differences in histopathological practices or incomplete diagnostic data.<br>2. Patient Demographics	4 - 11 Appendix A (16-20)

Section and Topic	Item #	Checklist item	Location where item is reported (page no.)
		• Age Range: 14 to 87 years; Mean = 52.5 years, SD = 16.97<br>• Gender Distribution: 58.2% female, 41.8% male<br>• No statistically significant differences in age between genders for any tumor type (all p-values > 0.05).<br>• Weak correlation between age and tumor type (r ≈ 0.13)<br>Conclusion: Age and gender do not significantly explain heterogeneity in outcomes.<br>3. Tumor Location<br>• Predominantly found in:<br>o Right atrium (25.5%)<br>o Right ventricle (21.8%)<br>o Mitral valve (14.5%)<br>• Variability in location may impact clinical presentation and treatment outcomes, contributing to study heterogeneity.<br>4. Clinical Presentation<br>• 21.8% asymptomatic (incidental discovery)<br>• Others presented with:<br>o Dyspnea (14.5%)<br>o Chest pain (10.9%)<br>o Rare symptoms: syncope, stroke, palpitations<br>• Symptom variability introduces clinical heterogeneity in case identification and management decisions.<br>5. Tumor Size<br>• Mean = 3.76 cm; Range: 0.68–11.05 cm<br>• Tumor size correlated with some clinical severity, but not consistently across all cases.<br>6. Diagnostic Modality<br>• Echocardiography used in 81.8% of cases — consistent modality<br>• CT (12.7%) and Chest X-ray (5.5%) used selectively<br>• Diagnostic tool used did not significantly affect outcomes, but may impact initial case recognition and detailed characterization.<br>7. Treatment Approaches<br>• Surgical resection in 87.3% of cases<br>• Non-surgical or biopsy-only in 12.7%<br>• Negative correlation between surgical treatment and tumor size reduction post-treatment (r = -0.51)<br>• Variability in whether surgery was chosen contributes to treatment-related heterogeneity.<br>8. Follow-Up Duration	

<table>
<tr><th>Section and Topic</th><th>Item #</th><th>Checklist item</th><th>Location where item is reported (page no.)</th></tr>
<tr><td></td><td></td><td>Highly variable: from 1 month to 14 years
19 cases had unspecified follow-up
Positive correlation between follow-up duration and stable disease (r = +0.51)
Inconsistent follow-up durations limit assessment of long-term outcomes and recurrence, introducing heterogeneity.
Statistical Analysis on Heterogeneity
T-tests: No significant gender-based age differences by tumor type
Correlation matrix:
Weak or no correlations for age vs. tumor type
Moderate correlations in follow-up vs. outcome variables
The study acknowledges significant heterogeneity in: histological reporting, patient demographics, tumor localization, symptomatology, treatment choice, follow-up duration
However, no single factor alone explains all variability. Rather, the combined effect of clinical, diagnostic, and institutional practices contributes to the observed heterogeneity in outcomes.</td><td></td></tr>
<tr><td></td><td>20d</td><td>While formal sensitivity analyses are absent, a few elements indirectly address robustness:
Correlation Analysis: explored relationships between variables (e.g., follow-up duration and stable disease; surgery and tumor size reduction). These findings offer some insight into how different factors may or may not influence outcomes
Comparative Statistics: T-tests used to compare age distributions by gender and tumor type. No statistically significant differences found, supporting the stability of demographic patterns
Subgroup Trends: outcomes compared across tumor subtypes, gender, and anatomical location. While this wasn't labeled as "sensitivity analysis," it does test the consistency of patterns across subsets</td><td>4 - 11
Appendix A
(16-20)</td></tr>
<tr><td>Reporting biases</td><td>21</td><td>Risk of Bias Due to Missing Results (Reporting Bias)
We evaluated the potential for reporting bias and missing results in each of the major syntheses included in this review, in accordance with PRISMA 2020 guidelines. Due to the nature of the study—a systematic review of case reports and small series—several areas of potential bias were identified:
1. Tumor Subtype Classification
Risk Level: Moderate
Assessment: 12 out of 55 cases (21.8%) were classified as “not specified” for tumor subtype. This proportion suggests moderate risk of selective reporting or insufficient histological detail in original reports. The missing classification data limits the completeness and accuracy of subtype-specific analyses.
Impact: Reduces confidence in exact subtype prevalence but does not invalidate the observed predominance of cavernous hemangiomas.
2. Follow-Up Duration and Outcomes
Risk Level: High
Assessment: Follow-up duration was not reported in 19 cases (34.5%), and recurrence status was missing in 18 cases (32.7%). These omissions represent a substantial gap in outcome data, likely due to selective follow-up or publication of early postoperative results</td><td>4 - 11
Appendix A
(16-20)</td></tr>
</table>

Section and Topic	Item #	Checklist item	Location where item is reported (page no.)
		without longitudinal monitoring.<br>• Impact: Introduces high risk of bias in the synthesis of long-term outcomes and recurrence rates. Reported low recurrence may be inflated by incomplete data.<br>3. Clinical Presentation and Symptoms<br>• Risk Level: Low<br>• Assessment: Nearly all included studies reported symptom status, including asymptomatic findings. The diversity of symptoms documented suggests minimal selective reporting in this category.<br>• Impact: Low risk of bias; results regarding symptomatic vs. incidental presentation are likely reliable.<br>4. Tumor Size<br>• Risk Level: Low to Moderate<br>• Assessment: Tumor size was reported in 52 of 55 cases (94.5%). The small number of missing entries limits risk, though underreporting of dimensions in cases diagnosed solely by echocardiography or post-mortem examination cannot be ruled out.<br>• Impact: Minimal impact on central tendency statistics; results remain robust.<br>5. Surgical Treatment and Management<br>• Risk Level: Low<br>• Assessment: Management strategy (surgical, conservative, or biopsy) was clearly reported in 100% of cases. There was no evidence of selective omission of non-surgical cases, which might bias toward curative outcomes.<br>• Impact: Low risk of bias; surgical trends are reflective of real-world practice.<br>6. Diagnostic Modality<br>• Risk Level: Low<br>• Assessment: All included cases documented the imaging modality used for diagnosis, with echocardiography being the most common. There is no indication of modality reporting bias.<br>• Impact: Low risk; imaging data synthesis is considered reliable.<br>7. Co-occurrence with Other Tumors<br>• Risk Level: Moderate<br>• Assessment: Coexisting tumors or systemic conditions were reported in a minority of cases. However, such information may have been omitted from reports deemed clinically irrelevant or where a cardiac hemangioma was incidental.<br>• Impact: Potential underestimation of syndromic or systemic associations.<br><br>While most syntheses showed low to moderate risk of bias, the greatest vulnerability lies in missing follow-up data and recurrence reporting, which impairs long-term outcome assessment. These limitations should be addressed in future studies through standardized reporting protocols and inclusion of longitudinal data.	
Certainty of evidence	22	We assessed the certainty (or confidence) in the body of evidence for each major outcome using a modified GRADE framework, which considers the nature of the evidence base (case reports and small series), risk of bias, consistency, directness, precision, and publication bias. Given the	4 - 11<br>Appendix A

Section and Topic	Item #	Checklist item	Location where item is reported (page no.)
		observational design and inherent limitations of case-based data, all outcomes started at low certainty, with possible upgrades or downgrades applied based on the criteria below.<br>1. Tumor Subtype Distribution<br>• Outcome: Cavernous hemangioma is the most common subtype.<br>• Certainty Level: Moderate<br>• Justification: Despite 12 cases classified as "not specified," the predominance of cavernous subtype is consistent with historical literature and across time. Data are direct and relatively consistent across sources.<br>• Downgrade: For incomplete reporting.<br>• Upgrade: For large effect size and consistency across multiple reports.<br>2. Symptomatology<br>• Outcome: 21.8% asymptomatic; dyspnea most common symptom.<br>• Certainty Level: Moderate<br>• Justification: Symptom status was reported in nearly all cases. Clinical presentation is heterogenous but well documented.<br>• Downgrade: Slight imprecision due to symptom subjectivity.<br>• Upgrade: Large, consistent effects observed (e.g., dyspnea in symptomatic patients).<br>3. Tumor Location<br>• Outcome: Right atrium and right ventricle are most commonly involved.<br>• Certainty Level: High<br>• Justification: Near-complete data reporting and consistent findings across cases and literature support a high level of confidence in this pattern.<br>4. Tumor Size<br>• Outcome: Mean tumor size 3.76 cm; wide variability.<br>• Certainty Level: Moderate<br>• Justification: Most cases included measurable data, but some size reports lacked precision or used inconsistent measurement formats.<br>• Downgrade: For heterogeneity in measurement and reporting.<br>5. Diagnostic Modality<br>• Outcome: Echocardiography used in 81.8% of cases.<br>• Certainty Level: High<br>• Justification: Imaging modality was clearly reported in all cases; strong, consistent evidence supports echocardiography as the primary diagnostic tool.<br>6. Surgical Management<br>• Outcome: 87.3% of cases underwent surgical excision.	(16-20)

Section and Topic	Item #	Checklist item	Location where item is reported (page no.)
		• Certainty Level: High<br>• Justification: Treatment data are complete and consistent. Strong consensus on surgery as the main intervention provides high confidence.<br>7. Recurrence After Surgery<br>• Outcome: 0% recurrence in cases with reported follow-up.<br>• Certainty Level: Low<br>• Justification: While recurrence was rarely observed, 33% of cases lacked recurrence status, and follow-up durations varied widely.<br>• Downgrade: For serious risk of reporting bias and imprecision.<br>• Note: This finding is plausible but not definitive.<br>8. Follow-Up Duration and Disease Stability<br>• Outcome: Stable disease in long-term monitored cases.<br>• Certainty Level: Low<br>• Justification: High variability in follow-up reporting and duration. Although stable outcomes were observed, absence of uniform surveillance limits certainty.<br>9. Co-Occurrence with Other Tumors<br>• Outcome: Rare association with liver hemangiomas, cancers, hematologic disorders.<br>• Certainty Level: Very Low<br>• Justification: Reporting was inconsistent, and incidental findings may have been underreported. Data insufficient to draw reliable conclusions.<br>The certainty in the evidence is highest for diagnostic modality, tumor location, and surgical treatment trends. However, conclusions regarding long-term outcomes, recurrence, and tumor associations are limited by missing or inconsistent data, highlighting the need for standardized reporting in future studies.	
**DISCUSSION**			
Discussion	23a	This systematic review provides a contemporary synthesis of 55 reported cases of cardiac hemangiomas over the past five years, offering valuable insight into their clinical presentation, histopathological features, diagnostic methods, and treatment outcomes. Although cardiac hemangiomas remain rare, the increasing number of reported cases—especially in 2023 and 2024—likely reflects improvements in imaging technologies, greater clinical awareness, and expanded case publication platforms, rather than a true rise in incidence.<br>The findings confirm longstanding observations in the literature, notably the predominance of cavernous hemangiomas as the most frequent subtype. This aligns with previous large-scale reviews, including Li et al. (2015), which similarly identified cavernous forms as the most commonly encountered histology among over 200 cases. The demographic profile observed—mean age of 52.5 years and slight female predominance—mirrors trends described in earlier reports but also highlights the wide age range of affected individuals, emphasizing the need to maintain diagnostic vigilance across all age groups.<br>Consistent with prior evidence, the right atrium and right ventricle emerged as the most commonly involved cardiac structures. The clinical manifestations varied, ranging from asymptomatic cases (21.8%) to those presenting with dyspnea, chest pain, and more severe complications such as arrhythmias and stroke—an observation that underlines the clinical heterogeneity of these tumors. Such variability supports earlier assertions that tumor size and anatomical location—not histological subtype—are the primary determinants of clinical significance.	12 – 14

Section and Topic	Item #	Checklist item	Location where item is reported (page no.)
		Importantly, this review reinforces the role of echocardiography as the cornerstone diagnostic tool, used in over 80% of cases. This aligns with established cardiology practice, where transthoracic and transesophageal echocardiography are first-line modalities for intracardiac mass detection. CT and MRI remain essential adjuncts for preoperative planning, especially in complex or ambiguous cases.<br>Surgical excision remains the mainstay of treatment, performed in 87.3% of cases. The absence of reported recurrences in cases with documented follow-up supports its curative potential. However, significant variability in follow-up duration and a high rate of unreported recurrence status (32.7%) temper the strength of this conclusion. This highlights a persistent gap in the literature—long-term surveillance data remain scarce, limiting our ability to fully assess recurrence risk or late complications.<br>Compared to older reviews that often included just a handful of cases over decades (e.g., Brizard et al., 1993), the current review demonstrates the growing feasibility of aggregating larger datasets. Nevertheless, the continued reliance on case reports and small series introduces inherent limitations such as reporting bias, inconsistent terminology, and selective outcome documentation. These limitations constrain the strength of pooled conclusions and underscore the need for standardized diagnostic criteria, histopathological classification, and follow-up protocols.<br>In summary, this review reinforces previous findings while contributing updated epidemiological and clinical insights. Cardiac hemangiomas, though rare, should be considered in the differential diagnosis of cardiac masses, particularly in the right atrium or ventricle. Echocardiography remains the most practical diagnostic approach, and surgery offers excellent outcomes when complete resection is achieved. Going forward, multicenter registries and prospective studies are needed to refine classification systems, optimize treatment algorithms, and evaluate long-term outcomes more reliably.	
	23b	While this systematic review provides a comprehensive synthesis of recent case reports and series on cardiac hemangiomas, several important limitations inherent to the available evidence should be acknowledged:<br>1. Study Design and Evidence Level<br>The review is based entirely on case reports and small case series, which represent the lowest tier of clinical evidence. These types of studies are highly susceptible to selection bias, reporting bias, and lack of generalizability. There were no randomized controlled trials or large observational studies available, limiting the ability to draw definitive conclusions or establish causality.<br>2. Incomplete and Inconsistent Reporting<br>A substantial proportion of included studies lacked critical data:<br>• Tumor subtype was unclassified in 12 cases (21.8%).<br>• Follow-up duration was unreported in 19 cases (34.5%).<br>• Recurrence outcomes were missing in 18 cases (32.7%).<br>This inconsistency compromises the robustness of synthesized outcomes, particularly for long-term prognosis and recurrence patterns. It also limits the depth of subgroup analysis and impairs the reliability of meta-synthesis.<br>3. Lack of Standardized Terminology and Diagnostic Criteria<br>The absence of a standardized classification system for cardiac hemangiomas leads to variability in how tumors are described and categorized. Some authors used general terms such as “vascular tumor” or “cardiac mass,” without confirming histological subtypes. Diagnostic approaches, criteria for surgical intervention, and follow-up strategies varied widely, further contributing to heterogeneity across studies.<br>4. Publication and Reporting Bias<br>There is likely a significant publication bias favoring the reporting of unusual, symptomatic, or surgically treated cases. Asymptomatic or conservatively managed hemangiomas may be underrepresented in the literature. Additionally, positive outcomes may be preferentially published, while complicated or recurrent cases may be less likely to appear in the literature.<br>5. Geographical and Institutional Bias	12 – 14

Section and Topic	Item #	Checklist item	Location where item is reported (page no.)
		Most included cases originate from specialized cardiovascular or academic centers with advanced imaging and surgical capabilities. This introduces a potential referral and institutional bias, as patients with complex or operable tumors are more likely to be published. The experience and resources available in these settings may not be generalizable to broader or resource-limited clinical environments.<br>6. Limited Long-Term Outcome Data<br>Despite surgical resection being the dominant treatment, few cases provided long-term follow-up beyond 12 months. Only three cases exceeded 5 years of follow-up. This paucity of longitudinal data limits understanding of potential late recurrences, complications, or mortality related to cardiac hemangiomas or their treatment.<br>Collectively, these limitations highlight the challenges of synthesizing high-certainty evidence for a rare condition based on isolated clinical reports. While valuable insights have emerged, particularly regarding diagnostic trends and surgical outcomes, the findings must be interpreted with caution. Future research should prioritize multicenter data registries, standardized reporting frameworks, and prospective studies to improve the quality, consistency, and applicability of the evidence base for cardiac hemangiomas.	
	23c	While this systematic review aimed to comprehensively assess recent literature on cardiac hemangiomas, several methodological limitations related to the review process itself should be acknowledged:<br>1. Single-Database Search Strategy<br>The literature search was conducted exclusively through PubMed, which, although comprehensive for biomedical research, may have excluded relevant studies indexed in other databases such as Embase, Scopus, Web of Science, or regional/non-English databases. This introduces a risk of publication selection bias.<br>2. Exclusion of Non-English and Grey Literature<br>Only English-language studies were included. Non-English publications, theses, conference abstracts, and other forms of grey literature were not assessed. As a result, the review may not fully capture all available global evidence, especially from countries with a high burden of cardiovascular disease but limited English-language publication rates.<br>3. No Formal Risk of Bias or Quality Appraisal Tools Applied<br>Although descriptive quality assessment was implicitly performed, the review did not employ standardized critical appraisal tools. The absence of these structured assessments may reduce transparency and rigor in evaluating the reliability of individual studies.<br>4. Narrative Rather than Quantitative Synthesis<br>Due to the heterogeneity and descriptive nature of the included studies, the review relied on narrative synthesis rather than meta-analysis. While appropriate for the data type, this approach limits the ability to estimate pooled effect sizes or perform statistical comparisons, potentially reducing the review's impact and precision.<br>5. Potential for Reviewer Bias<br>Given the absence of a pre-registered review protocol (e.g., PROSPERO registration), there is a risk of reviewer-driven decisions regarding inclusion/exclusion criteria, outcome prioritization, and synthesis methods.<br>These methodological limitations, while common in rare disease reviews, underscore the importance of structured, transparent, and reproducible review protocols. Future reviews would benefit from broader search strategies, independent review teams, use of formal bias assessment tools, and protocol pre-registration to enhance the rigor and credibility of findings.	12 – 14

Section and Topic	Item #	Checklist item	Location where item is reported (page no.)
	23d	Implications for Clinical Practice<br><br>The findings of this systematic review underscore several critical considerations for clinicians managing patients with suspected or confirmed cardiac hemangiomas:<br><br>• Maintain High Suspicion in Incidental Cardiac Masses: Despite their rarity, cardiac hemangiomas should be included in the differential diagnosis of intracardiac tumors, particularly those involving the right atrium or right ventricle. The wide age distribution and frequent asymptomatic presentation highlight the need for vigilance during routine imaging and cardiac evaluations.<br><br>• Echocardiography as First-Line Imaging: The overwhelming reliance on echocardiography (81.8%) as the primary diagnostic tool supports its role as the standard first-line investigation. Transthoracic and transesophageal echocardiography should be prioritized for early identification, with CT or MRI used as adjuncts for surgical planning or anatomical clarification.<br><br>• Surgery as Definitive Therapy: With 87.3% of patients undergoing surgical resection and virtually no documented recurrences in those with adequate follow-up, surgical excision should remain the treatment of choice in eligible patients. However, careful patient selection is needed, especially in asymptomatic cases or those with high operative risk.<br><br>• Individualized Follow-Up Plans Needed: Given the lack of standardized follow-up protocols and the variability in recurrence reporting, clinicians should adopt an individualized, risk-based follow-up strategy, particularly in non-surgical or incompletely resected cases.<br><br>Implications for Policy and Standardization<br><br>• Need for Structured Reporting Guidelines: The review highlights a significant lack of standardization in case reporting, particularly with regard to histological classification, follow-up duration, and recurrence monitoring. Development and adoption of standardized reporting templates or checklists for rare cardiac tumors would improve consistency and data quality across case reports.<br><br>• Promote Rare Tumor Registries: Health systems and cardiovascular centers should be encouraged to contribute to multicenter rare tumor registries, enabling more robust data aggregation, outcome tracking, and real-time learning. Such initiatives can accelerate knowledge generation for rare diseases and support evidence-informed decision-making.<br><br>• Integration of Rare Tumor Guidelines into Cardiac Protocols: National cardiology societies and surgical associations may consider integrating rare tumor considerations—such as diagnostic pathways and surgical approaches—into broader guidelines for cardiac mass evaluation and management.<br><br>Implications for Future Research<br><br>• Multicenter Prospective Studies: To overcome the limitations of single-institution case reports, prospective, multicenter studies are needed to assess long-term outcomes, recurrence rates, and comparative treatment effectiveness.<br><br>• Molecular and Genetic Profiling: The etiology of cardiac hemangiomas remains poorly understood. Molecular, genetic, and biomarker studies may help elucidate potential pathogenic mechanisms or syndromic associations and guide targeted therapies in the future.	12 – 14

PRISMA 2020 Checklist

Section and Topic	Item #	Checklist item	Location where item is reported (page no.)
		• Longitudinal Follow-Up Studies: Given the lack of consistent long-term follow-up, future research should focus on longitudinal surveillance of surgically and non-surgically managed cases, enabling more accurate estimation of recurrence, stability, and patient quality of life over time.<br>• Comparative Effectiveness of Imaging Modalities: Additional research is needed to evaluate the diagnostic performance and prognostic value of different imaging modalities (e.g., cardiac MRI, PET-CT) in distinguishing hemangiomas from other cardiac tumors.	
**OTHER INFORMATION**			
Registration and protocol	24a	This systematic review was not registered in a prospective review registry such as PROSPERO.	
	24b	A formal review protocol was not prepared for this systematic review.	
	24c	This review was conducted without a formal protocol; therefore, no protocol amendments were applicable.	
Support	25	This review was supported by Victor Babes University of Medicine and Pharmacy Timișoara, which covered the publication-related costs.<br>Role of the Funder<br>The funder had no role in the following aspects of the review: study design, data collection, analysis, or interpretation, manuscript preparation, decision to submit the manuscript for publication<br>All views and conclusions expressed in this review are those of the authors and do not necessarily reflect the official policy or position of the supporting institution.	15
Competing interests	26	The authors declare no competing interests relevant to the content of this review.	15
Availability of data, code and other materials	27	The following materials related to this systematic review are not publicly available at this time:<br>• Template data collection forms<br>• Extracted data from included studies<br>• Data used for analyses<br>• Other materials (e.g., quality assessment tools, charts)<br>These materials were used internally by the authors during the review process and have not been deposited in a public repository.<br>However, the authors are committed to transparency and will provide access to these materials upon reasonable request by contacting the corresponding authors at streian.caius@umft.ro andrei.manzur@umft.ro.	
